# Dual-Energy CT in the Acute Setting: Bowel Trauma

**DOI:** 10.3389/fradi.2022.835834

**Published:** 2022-04-04

**Authors:** Tanche Jimmy Wang, Sarah Barrett, Ismail Ali, Faisal Khosa, Savvas Nicolaou, Nicolas Murray

**Affiliations:** Emergency Radiology Division, Vancouver General Hospital, University of British Columbia, Vancouver, BC, Canada

**Keywords:** dual-energy, CT, trauma, virtual monoenergetic imaging, virtual non-contrast imaging, virtual non-calcium imaging

## Abstract

Traumatic bowel and mesenteric injuries (TBMI) have significant morbidity and mortality. The physical examination is often limited and sometimes not feasible in the trauma patient. Multidetector CT (MDCT) detection of TBMI is challenging and can be life-saving. Dual-energy CT (DECT) utilizes iodine overlay, monoenergetic imaging, and metal artifact reduction to enhance the conspicuity of TBMI. DECT may improve the conspicuity of TBMI leading to increased diagnostic accuracy and confidence. The aim of the article is to review the state of the art and applications of DECT in bowel trauma.

## Introduction

### Dual-Energy CT Background

Dual-energy CT (DECT) utilizes CT acquisition of data at two different energy levels (1). Based on the differential attenuation of material at the two different energy levels, information on the composition of a given tissue can be accurately obtained. Despite DECT existing since the 1970s, use was limited due to limitation in acquisition times until recent advances in CT technology. DECT has been utilized in cardiothoracic, vascular, musculoskeletal, and neuro imaging (2–14). Abdominal applications have been utilized with a predominance on the solid organs, neoplasm, and inflammatory bowel disease (15–24). The application of DECT in emergency radiology and bowel trauma is a recent advancement, with only a few studies published to date. DECT enables the ability to obtain material-specific imaging with iodine overlay maps and virtual unenhanced images, as well as from virtual monoenergetic reconstructions to improve detection and confidence. This article will review the general principles of CT and DECT imaging, with emphasis on the clinical applications specific to bowel and mesenteric injuries.

### Dual-Energy CT Physical and Technical Considerations

The X-ray photons used in CT interact with matter by photoelectric or Compton scattering (25). Photoelectric is predominant at lower kilovoltage, which depends on the tissue density or atomic number resulting in high contrast and noise. Higher kilovoltage interaction results in predominantly Compton scattering, which yields lower attenuation, noise, and contrast. Conventional single-source CT uses a single polyenergetic photon beam with a set peak-energy level between 80 and 120 kVp to optimize contrast and noise.

Attenuation of material is dependent on the energy of the incoming photon beam, and the atomic number and density of the material. Each material has a specific attenuation property that varies at different peak-energy levels for a concentration to be kept constant. However, at a given peak-energy level, 2 different materials can demonstrate a similar attenuation depending on their respective concentration, which makes it impossible to separate them using conventional single-peak-energy CT imaging.

DECT simultaneously acquires images at low (80–90 kVp) and high (140–150 kVp) energy levels using a single source with rapid alternation between high and low energy levels, two sources and detectors pairs, or a single source with dual-layer detectors to acquire images at high and low energy levels.

DECT uses the attenuation signature of each material at different energy levels. This property enables differentiation of some materials with high atomic numbers such as calcium and iodine from low-atomic-number materials such as soft tissue structures made of carbon, oxygen, hydrogen, and nitrogen, which present little to no variation in attenuation within the range of energies used in diagnostic imaging. Voxel containing iodine and calcium can therefore be identified with precision, highlighted in material-specific images (iodine overlay images and calcium overlay images), or subtracted from the image to create the virtual non-contrast (VNC) or virtual non-calcium (VNCa) images.

Monoenergetic image reconstruction produces images that simulate an image that would be acquired with a pure single-energy photon beam and help improve iodine contrast and contrast between structures.

DECT possesses many benefits from diagnostic, cost, and radiation perspectives. DECT has been shown to add value to routine emergency department imaging by increasing diagnostic confidence, reducing follow-up studies and costs (26). DECT has no radiation dose penalty with the potential for dose reduction. Abdominal DECT can be performed without increased radiation dose or deteriorating image quality compared to dose-optimized single energy CT (27). Using virtual unenhanced images to replace true unenhanced images reduces radiation dose up to 47% with equivalent quality (28).

### Dual-Energy CT and Bowel Injury

Traumatic bowel and mesenteric injuries (TBMI) have an incidence of ~1% with significant morbidity and mortality from delays in diagnosis and prolonged hospitalization (29). CT abdomen and pelvis with intravenous contrast is the recommended study for suspected bowel trauma in major blunt trauma (30). Early recognition of bowel injury significantly decreases morbidity and mortality (31). The increasing use of CT in trauma has led to a trend toward conservative management in trauma patients. The physical examination is often limited and unreliable and may even not be feasible in trauma patients. A wide range of sensitivities and specificities exist for CT diagnosis of TBMI. Detection of TBMI in trauma patients even with the use of multi-detector CT is challenging, commonly with a paucity of imaging findings to support the diagnosis and the presence of subtle or indirect radiological signs, in addition to the often multiple concomitant distracting injuries. DECT significantly improves the conspicuity of ischemic bowel compared to conventional CT by increasing attenuation differences between ischemic and perfused segments on iodine overlay images and low-energy virtual monoenergetic imaging (32). DECT increases accuracy and confidence in the diagnosis of vascular and enteric extravasated contrast material in animal models of abdominopelvic trauma (33).

## Applications

### Dual-Energy CT Reconstructions

Dual-energy images are reconstructed after data acquisition, which can be time-consuming when performed manually. Automated reconstruction performed by post-processing software for all patients can streamline workflow and save time when DECT is critically needed. Different DECT reconstructions including virtual non-contrast images, iodine overlay images, and high- and low-energy monoenergetic images can be routinely automatically reconstructed for quick access as needed.

### Dual-Energy CT Increases Conspicuity of Hyperattenuating Free Fluid

**Figure 1:**
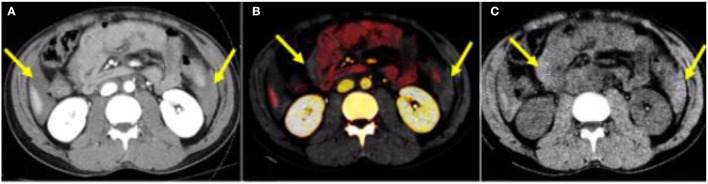
A 28-year-old male patient—after a motor vehicle accident. **(A)** Conventional mixed DECT image demonstrates free fluid in the abdomen (yellow arrows). **(B)** Iodine overlay images increase the conspicuity of free fluid by differentiating enhancing bowel from non-enhancing fluid (yellow arrows). **(C)** DECT VNC accentuates the hyperattenuating nature of the hemoperitoneum. DECT, dual-energy CT; VNC, virtual non-contrast.

**Figure 2:**
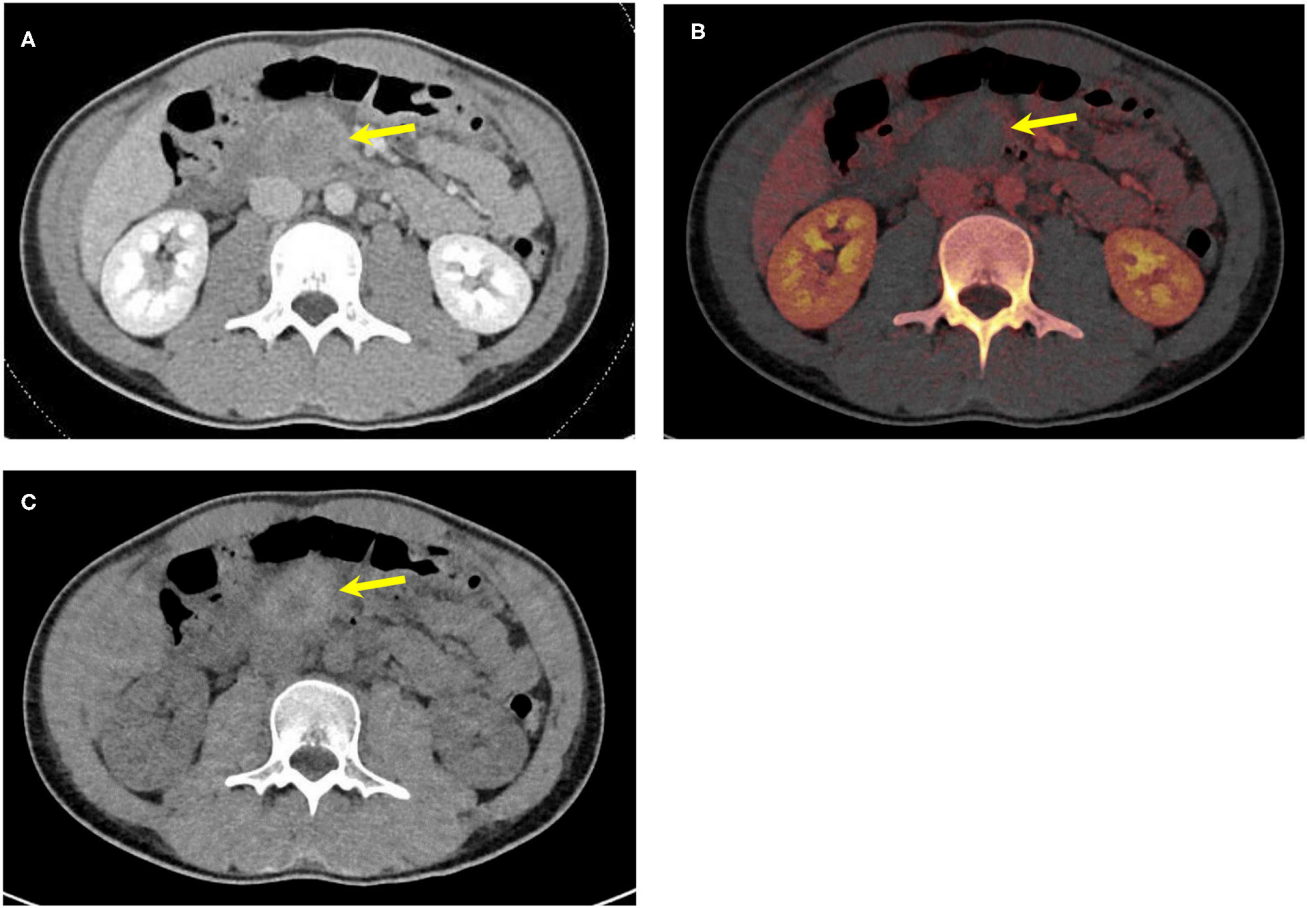
An 18-year-old male patient—hockey stick injury. **(A)** Conventional mixed DECT image demonstrates duodenal wall hematoma (yellow arrow). **(B)** Iodine overlay images increase the conspicuity of the duodenal hematoma by differentiating enhancing bowel from a non-enhancing duodenal hematoma (yellow arrow). **(C)** DECT VNC accentuates the hyperattenuating nature of the duodenal hematoma (yellow arrow). DECT, dual-energy CT; VNC, virtual non-contrast.

Identifying free fluid in the mesentery in between bowel loops can sometimes be a sign of mesenteric injury (34). Hyperdense fluid and hemoperitoneum in the mesentery can be difficult to discern from adjacent enhancing bowel loops considering their relatively similar attenuation, especially with a paucity of abdominal fat. Iodine overlay images demonstrate iodine-containing voxels as color-coded in red, therefore highlighting enhancing bowel loops and increasing conspicuity of non-enhancing free fluid adjacent to the bowel loops (Figure 1). DECT VNC images subtract the mucosal enhancement of the bowel loops and increase the hyperattenuating nature of the dense free fluid representing blood in the setting of trauma-related hemoperitoneum. DECT VNC may accentuate the hyperattenuating nature of acute bowel wall hematoma (Figure 2).

### Lack of Iodine Uptake in Injured Bowel

**Figure 3:**
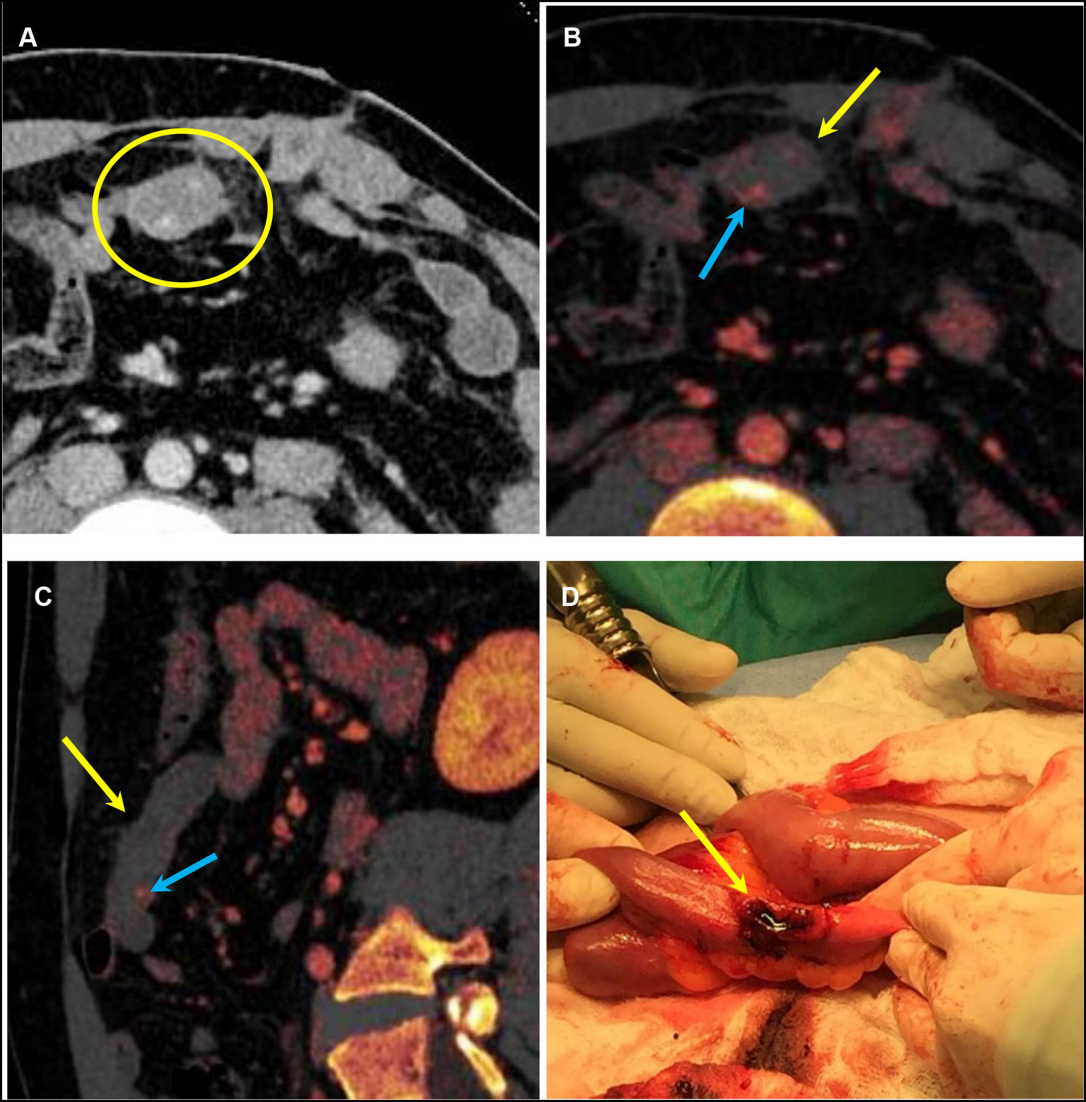
A 25-year-old male patient with a stab wound to the anterior abdomen. **(A)** Conventional mixed DECT image demonstrates mild thickening of a small bowel loop (yellow arrow). [**(B)** Axial and **(C)**] Sagittal oblique DECT iodine overlay images demonstrating an area of transmural lack of iodine uptake anteriorly (yellow arrows) and a small area of endoluminal contrast extravasation not seen on the conventional image (blue arrows) concerning a penetrating injury. **(D)** Intraoperative picture confirming the focal transmural small bowel perforation (yellow arrow). DECT, dual-energy CT.

**Figure 4:**
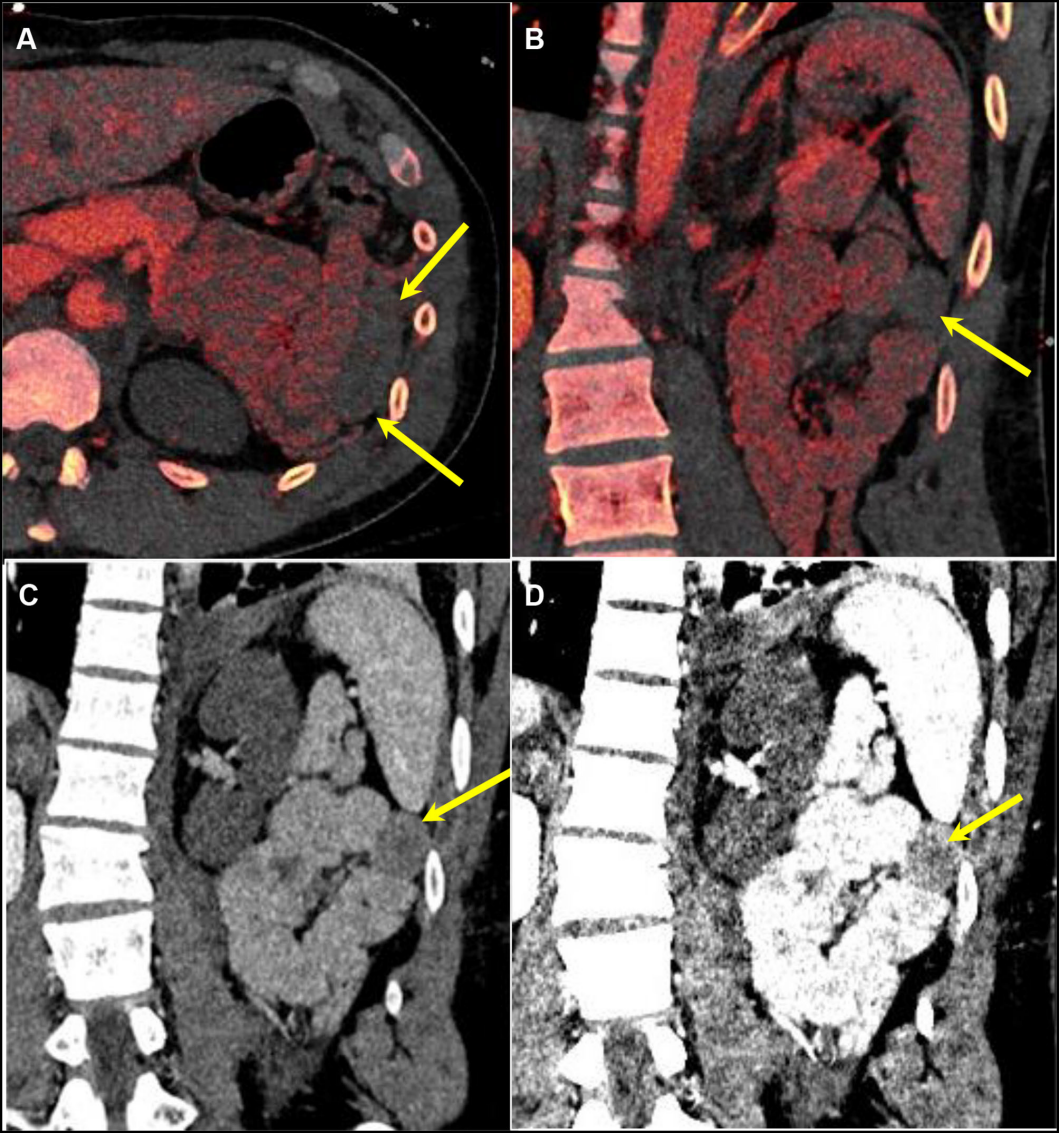
A 25-year-old male patient presenting after a high-speed motor vehicle accident. [**(A)** Axial and **(B)**] Coronal DECT iodine overlay images demonstrating decreased iodine uptake in a left upper quadrant small bowel loop compared to the adjacent loop (yellow arrows). DECT VMI reconstructions at **(C)** 70 keV (equivalent to a conventional CT at 120 kVp) and **(D)** 40 keV demonstrating a higher difference in attenuation between the normal and abnormal loops at a lower energy level. DECT, dual-energy CT; VMI, virtual monoenergetic images.

**Figure 5:**
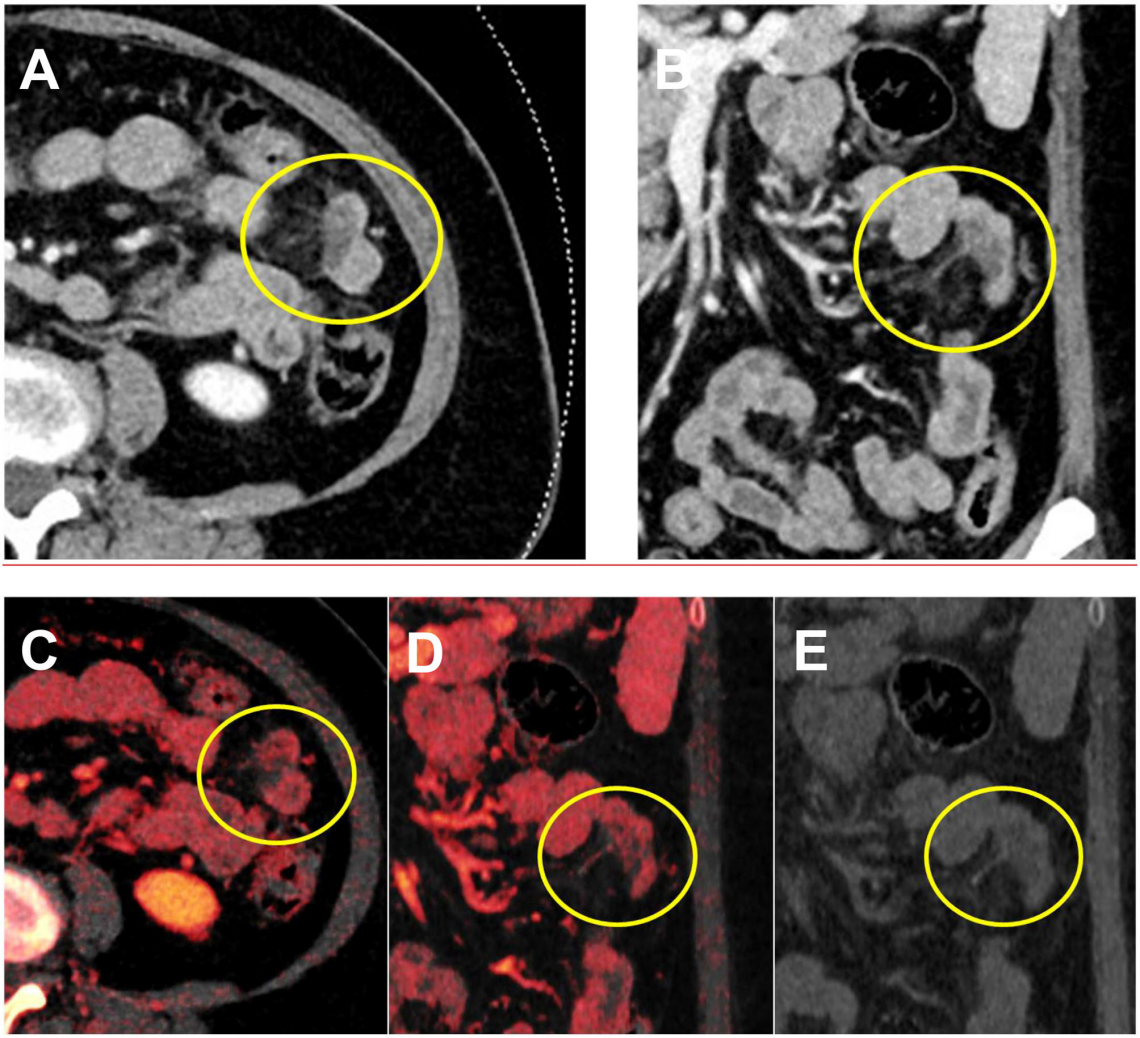
A 17-year-old male patient presenting with pain and vomiting after trauma during a basketball game. **(A,B)** Conventional CT demonstrates hypodensity in the bowel wall. **(C,D)** No internal iodine enhancement and uptake were confirmed using the iodine overlay map, confirming bowel wall hematoma. **(E)** On the virtual non-contrast image, the bowel wall remains hypodense.

Intramural hematoma results in circumferential or eccentric thickening of the bowel wall, which is an indication of blunt trauma of the bowel (31). Bowel injury can result in decreased enhancement and decreased iodine uptake on DECT. The iodine map and low-energy virtual monoenergetic images (VMI; for example, at 40–50 keV) increase conspicuity between enhancing and non-enhancing bowel to improve diagnostic accuracy as compared with conventional CT images alone (35). The iodine overlay map can help identify areas of decreased iodine uptake and increase detection and confidence (Figures 3, 4). The lower-keV DECT images also increase the attenuation of the normal enhancing bowel and accentuate the difference between bowel with decreased enhancement and the normally enhancing adjacent bowel. Post-traumatic bowel hematomas can present as a mass in the bowel wall, which can be confirmed using dual energy to demonstrate the absence of iodine uptake with accentuated attenuation on low-keV images (Figure 5).

### Metal Artifact Reduction

**Figure 6:**
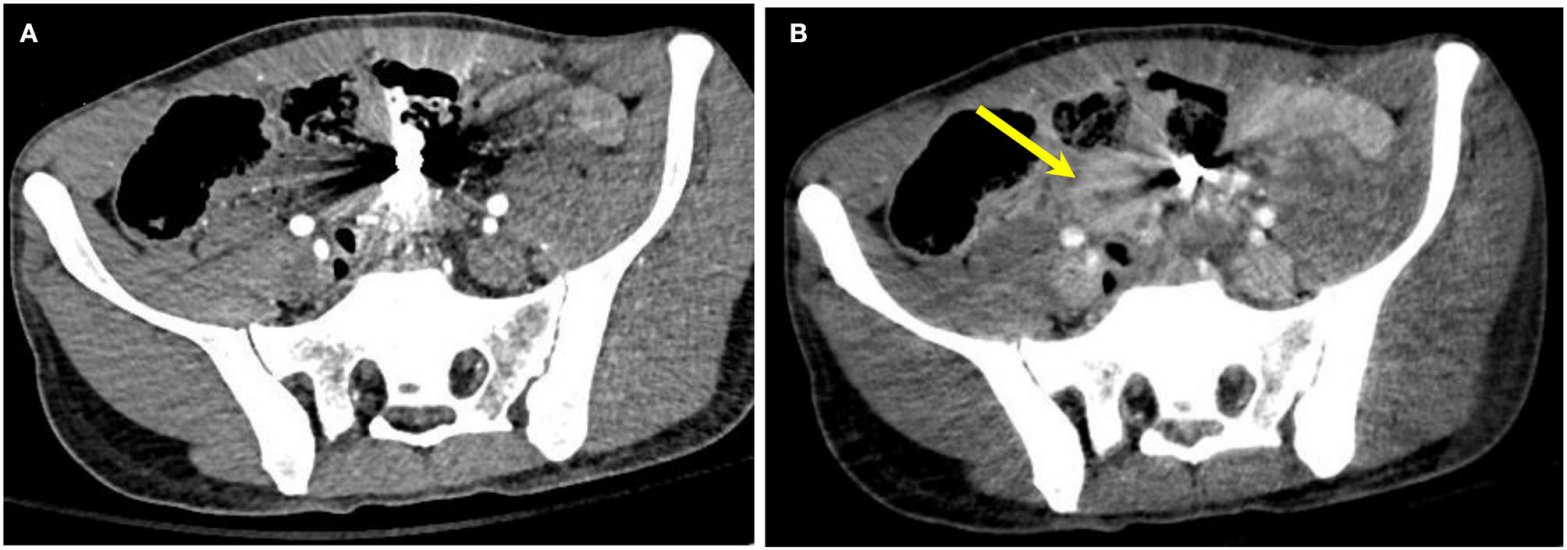
A 25-year-old man with a gunshot wound. **(A)** Conventional CT shows bullet fragments with streak artifacts. **(B)** Image 140 keV demonstrates decreased streak artifact revealing thickened bowel loops (arrow).

Penetrating bowel injury from gunshot or knife wounds can leave metal fragments within the abdominal cavity, with resulting streak artifacts limiting local assessment. VMI at high keV can significantly reduce streak artifacts and reveal underlying pathology (Figure 6) but are associated with a decreased soft-tissue contrast resolution. The optimal level of keV will vary depending on the cause and nature of the artifact, generally between 130 and 190 keV. A combination of monoenergetic reconstructions at higher energy levels and metal artifact reduction software (MARS) has been shown to reduce metal artifacts even further (36).

### Contrast Extravasation

**Figure 7:**
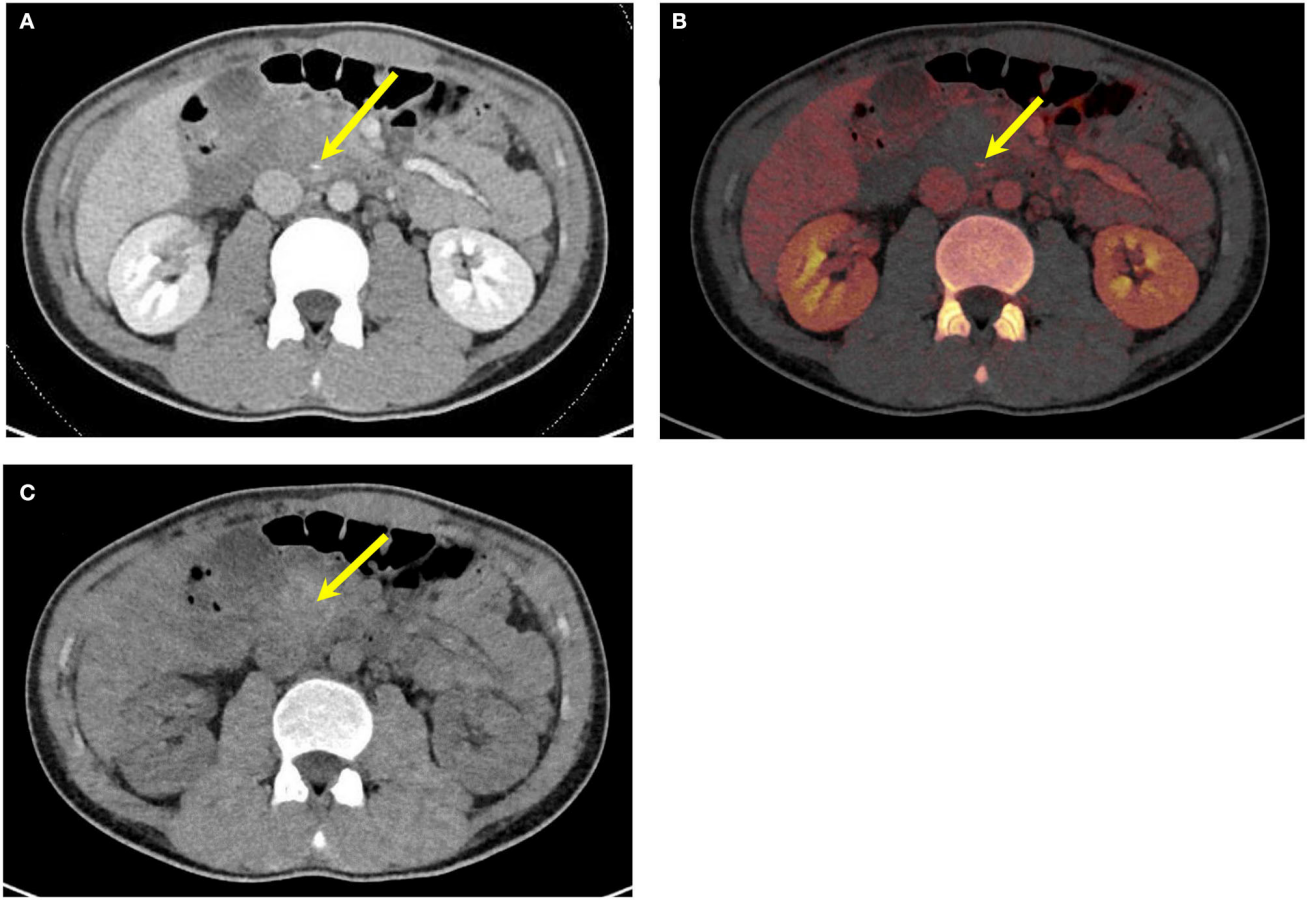
An 18-year-old male patient—hockey stick injury. **(A)** Conventional mixed DECT image demonstrates intraluminal active contrast extravasation (yellow arrow). **(B)** Iodine overlay images increase the conspicuity of the intraluminal contrast extravasation (yellow arrow). **(C)** DECT VNC—focus of contrast is not seen on DECT VNC, confirming the hyperdensity is contrast and not pre-existing on nonenhanced CT (yellow arrow). DECT, dual-energy CT; VNC, virtual non-contrast.

Bowel and mesenteric injuries can result in active contrast extravasation. Dual-energy iodine overlay can facilitate the detection of contrast extravasation. Low-keV VMI increase the attenuation of the iodinated contrast and increase the conspicuity of contrast extravasation. Intraluminal or extraluminal contrast extravasation can occur post-trauma with improved detection using dual energy (Figures 3, 7). The overall accuracy of identification of the source of extravasation increased from 78% with conventional CT to 92% with DECT (33).

### Foreign Body

Foreign bodies from ingestion, penetrating or incidental such as surgical gauze, can be assessed with virtual non-contrast images or VMI. Virtual non-contrast images can confirm that the foreign body is inherently radiodense and not due to intravenous contrast administration. VMI can enhance conspicuity for dense objects at lower-keV images with improved contrast resolution or reduce streak artifacts with higher-keV images (Figure 6).

## Conclusion

TBMI are uncommon but clinically significant and can result in significant morbidity and mortality. TBMI diagnosis is clinically challenging in trauma patients. CT findings of TBMI can be non-specific or subtle. DECT improves conspicuity, diagnostic confidence, and accuracy in TBMI.

## Author Contributions

All authors listed have made a substantial, direct, and intellectual contribution to the work and approved it for publication.

## Conflict of Interest

FK is the recipient of the American College of Radiology – Global Humanitarian Award (2021) and the Royal College of Physicians and Surgeons of Canada McLaughlin-Gallie Visiting Professor (2021). University of British Columbia (UBC) received a master's research agreement with Siemens Healthineers. The remaining authors declare that the research was conducted in the absence of any commercial or financial relationships that could be construed as a potential conflict of interest.

## Publisher's Note

All claims expressed in this article are solely those of the authors and do not necessarily represent those of their affiliated organizations, or those of the publisher, the editors and the reviewers. Any product that may be evaluated in this article, or claim that may be made by its manufacturer, is not guaranteed or endorsed by the publisher.
